# The longest diameter of tumor as a parameter of endoscopic resection in early gastric cancer: In comparison with tumor area

**DOI:** 10.1371/journal.pone.0189649

**Published:** 2017-12-20

**Authors:** Yoo Jin Um, Hae Won Kim, Da Hyun Jung, Jie-Hyun Kim, Jae Jun Park, Young Hoon Youn, Hyojin Park, Jong Won Kim, Seung Ho Choi, Sung Hoon Noh

**Affiliations:** 1 Department of Internal Medicine, Yonsei University College of Medicine, Seoul, Korea; 2 Gangnam Severance Hospital, Yonsei University College of Medicine, Seoul, Korea; 3 Department of Surgery, Yonsei University College of Medicine, Seoul, Korea; National Cancer Center, JAPAN

## Abstract

**Background and aim:**

Tumor burden is important to predict clinical behaviors of cancer such as lymph node metastasis (LNM). Tumor size has been used as a parameter of tumor burden such as indication of endoscopic resection in early gastric cancer (EGC) to predict LNM. Thus, we aimed to investigate whether tumor area can be more helpful to predict clinical behaviors than longest diameter of tumor in EGC.

**Patients and methods:**

3,059 patients who underwent gastrectomy for EGC were reviewed retrospectively. Tumor area was calculated by multiplying long and short diameter of the tumor in surgical specimen. Longest diameter means maximal longitudinal diameter of tumor in specimen. Clinicopathologic features were compared between longest diameter and area using area under receiver operating characteristic (AUROC) curves.

**Results:**

Longest diameter and area of tumor showed a strong correlation (correlation coefficient 0.859, *p*<0.01). The cutoff value for prediction of LNM was 20 mm of longest diameter of tumor and 270 mm^2^ of tumor area. There was no significant difference between longest diameter and area for prediction of LNM (AUC 0.850 vs. 0.848, respectively). In differentiated-type EGC and undifferentiated-type EGC, there was no significant difference between longest diameter and area for prediction of LNM. Among mucosal or submucosal cancer prediction value of LNM between longest diameter and area was not significantly different.

**Conclusion:**

Tumor area may not be more helpful to predict LNM than longest diameter in EGC. Therefore, the longest diameter of tumor may be sufficient as an indicator of tumor burden in EGC.

## Introduction

Early gastric cancer (EGC) is defined as confined to gastric mucosa or submucosa with low incidence of nodal metastasis[1], endoscopic resection (ER) is the curative treatment option for EGC. Curative ER means en bloc resection, negative horizontal and vertical margins, no lymphovascular invasion (LVI), and that meet the absolute or expanded indication[2]. However, following pathological evaluations, any ER does not satisfy these criteria is considered a noncurative resection. The prognosis of EGC tends to be good, but some patients still require additional surgery after ER. Although nodal status does not affect the designation of EGC, lymph node metastasis (LNM) is one of the most meaningful prognostic factors. The incidence of LNM in EGC ranges from 0% to 3% for intramucosal cancer and from 11% to 20% for submucosal cancer[3, 4]. In some large-scale studies from Japan and Korea, the overall survival rate of lymph node-positive EGC fell to 70%-80%[5], and the local recurrence rate after ER of EGC ranges from 0.4% to 3.7% [6] and the metachronous recurrence rate from 2.7% to 14.0%[7]. Thus, the LNM prediction before ER for EGC plays an important role in the prognosis of EGC. Jung et al.[8] reported that the elevated type was significantly associated with LNM in differentiated-type EGC. They represented that clinical behaviors vary by the endoscopic gross appearance of EGC and tumor burden is predicted by endoscopic gross appearance. They suggested tumor burden is important to predict clinical behaviors of cancer such as LNM. In order to represent tumor burden of EGC, usually the longest diameter of tumor; maximal longitudinal diameter of tumor was measured[9, 10]. The generally accepted indications of ER for EGC, based Gotoda’s data, are used the longest diameter of tumor [2]. However, several studies reported that the clinical significance of tumor area or volume measured by endoscopic ultrasound in esophageal cancer and EGC[11, 12]. Therefore, we aimed to identify whether tumor area can be more helpful to predict clinical behaviors than the longest diameter of tumor in EGC. If tumor area is more helpful to predict clinical behaviors, we thought it would be able to use to predict LNM in ER.

## Materials and methods

### Patients and definition

We retrospectively reviewed clinicopathologic findings of 3,059 patients underwent gastrectomy for EGC from January 2005 to December 2012 at Severance and Gangnam Severance Hospital. These data included the longest diameter of tumor, the presence of LNM, depth of invasion, and histologic grade using pathologic specimen after surgical resection. All surgical specimens were routinely fixed in 10% formalin and were then serially sectioned at 5-mm intervals, embedded in paraffin blocks, and stained with hematoxylinand eosin. The depth of tumor invasion was then evaluated with lymphovascular involvement and degree of differentiation.LNM was identified using hematoxylinand eosin staining. We defined the longest diameter of tumor means maximal longitudinal diameter of the tumor in pathologic specimen and the tumor area was calculated by multiplying the longestdiameter and its perpendicular short diameter of the tumor in specimen. The study was conducted according tothe provision of the Declaration of Helsinki. This study was approved by the ethics committee and written informed consent was obtained from all subjects.

### Comparison of LNM prediction value by the longest diameter and area of tumor

First, we analyzed the relationships between the longest diameter and area of tumor in EGC and measured cut-off values of the longest diameter and area of tumor in EGC. And then, we compared the LNM prediction value of the longest diameter and area of tumor in total EGC. In addition, we performed subgroup analysis for the LNM prediction value of the longest diameter and area of tumor according to the histologic classification and the tumor depth.

### Statistical analysis

Continuous variables are reported as means ± standard deviation (SD). The relationship between longest diameter and area of tumor analyzed by pearson correlation coefficient. It is a measure of the linear correlation between two variables that is defined as the covariance of the variables divided by the product of their standard deviations. The predictive performance of the longest diameter and area of tumor for lymph node metastasis was evaluated using receiver operating characteristic (ROC) curves. The Youden index is the difference between the true positive rate (sensitivity, %)and the false positive rate (1-specificity, %)[13]. Finding the point on the ROC curve that maximizes the Youden index provides an optimal cutoff value that is independent of the prevalence rate. Using this index, we get cut-off values of longest diameter and area of tumor in EGC. The cutoff value was defined the highest sum of the sensitivity and specificity. Comparisons of AUCs were performed using the method described by DeLong et al[14] for correlated data. Clinicopathologic features were compared between tumor area and the longest diameter of tumor using area under receiver operating characteristic (AUROC) curves. Analyses were undertaken using SPSS ver. 20.0 for Windows (SPSS, Chicago, IL, USA), SAS ver. 9.2 (SAS Institute, Cary, NC, USA) and MedCalc ver. 12.7.0 (MedCalc Software, Ostend, Belgium). Two tailed p-value <0.05 indicated statistical significance.

## Results

### Baseline characteristics of study group

Table 1 shows the baseline characteristics of total enrolled patients. The median age of total patients was 57 years (range, 23–86 years), and the male to female ratio was 1.88:1. The median the longest diameter was 26mm (range,1-220mm) and the median tumor area was 634mm^2^(range, 1-16900mm^2^). Among 3,059 patients LNM was observed in 321 patients (10.4%). In addition, we analyzed the associated factors for the LNM in total EGC patients by multivariate analysis at Table 2.

**Table 1:** Baseline demographic factors of patients.

	Value (n, %)
Age (mean ± SD, years)	57.0±11.0
Sex	
Male	1995 (65.3)
Female	1064 (34.7)
The longest diameter (mm,mean±SD)	26.0±15.0
Area (mm, mean±SD)	634±971
Location	
Upper 1/3	322 (10.5)
Middle 1/3	530 (17.3)
Lower 1/3	2207 (72.2)
Depth of invasion	
Mucosa	1578(51.5)
Submucosa	1481(49.5)
Japanese classification	
Differentiated	717(23.4)
Undifferentiated	2342(76.6)
Lauren classification	
Intestinal	1702 (55.6)
Diffuse	1184 (38.7)
Mixed	173 (5.7)
LN metastasis	
Positive	321(10.4)

**Table 2:** Multivariate analysis of risk factors for the lymph node metastasis in EGC.

	Odds ratio	*P-value*
	(95% confidence interval)	
The longest diameter	1.027 (1.015–1.040)	<0.001
Gross appearance	-	NS
Ulceration +	1.625(1.276–2.069)	<0.001
Lauren classification		
Diffuse	0.525 (0.335–0.823)	0.005

### Analysis of LNM prediction value between the longest diameter and area of tumor in total EGC

**Fig 1:**
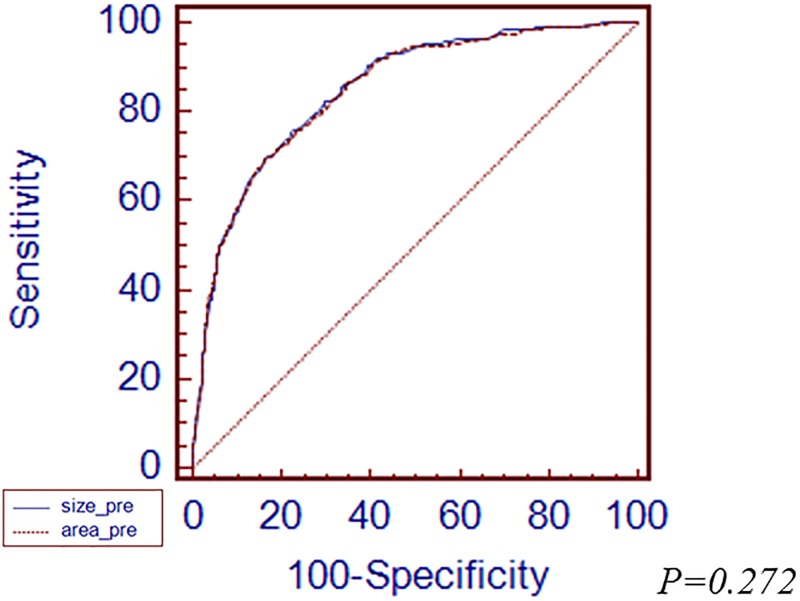
Comparison of receiver operating characteristic (ROC) curves demonstrating the prediction power of lymph node metastasis (LNM) between the longest diameter and area of tumor in early gastric cancer (EGC).

The longest diameter and area of tumor showed a strong correlation (Pearson correlation coefficient 0.859, *p<0*.*01*,S1 Fig). When analyzed among the total EGC patients, the cutoff value for prediction of LNM was 20 mm of the longest diameter and 270 mm^2^ of tumor area. If the value of the longest diameter or tumor area was lower than the cutoff value, we considered that it showed the low probability of LNM.ROC curve was made using the cut off value, the AUROC value of the longest diameter was 0.850 and tumor area was 0.848(Fig 1). There was no significant difference between the longest diameter and area of tumor for prediction of LNM.

### Subgroup analysis by histologic type and depth of invasion

**Fig 2:**
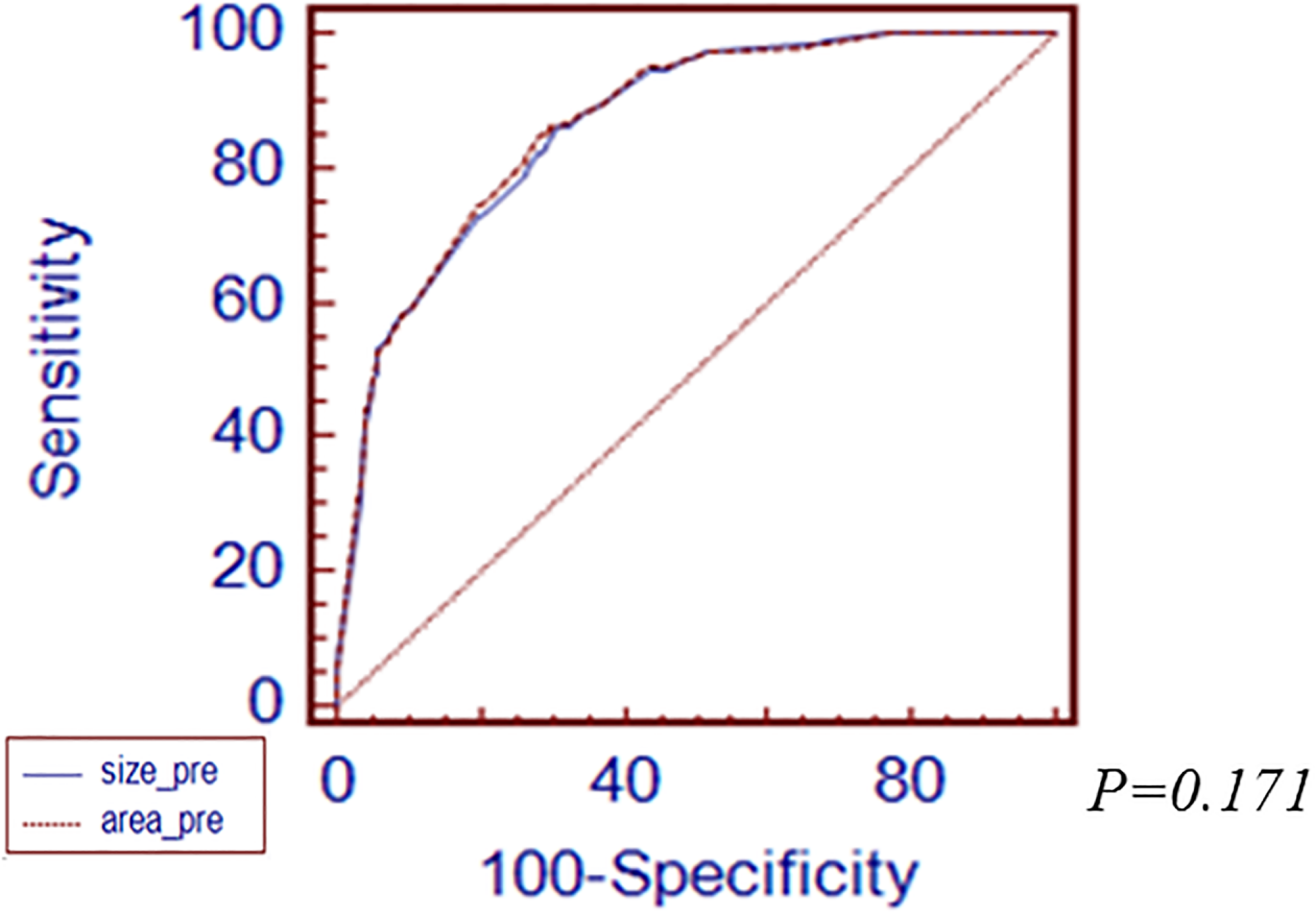
Comparison of ROC curves demonstrating the prediction power of LNM between the longest diameter and area of tumor in differentiated-type EGC.

**Fig 3:**
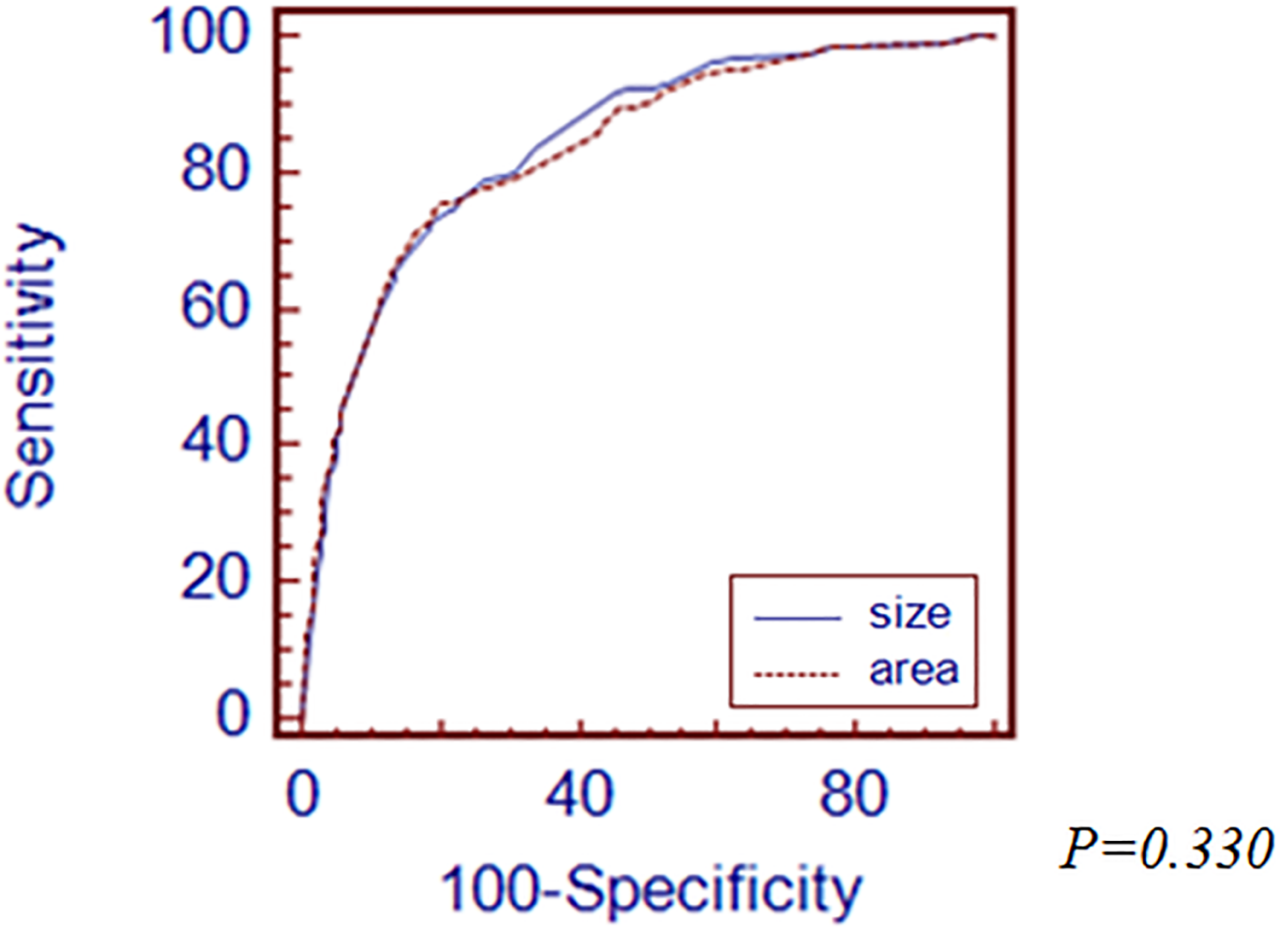
Comparison of ROC curves demonstrating the prediction power of LNM between the longest diameter and area of tumor in undifferentiated-type EGC.

**Fig 4:**
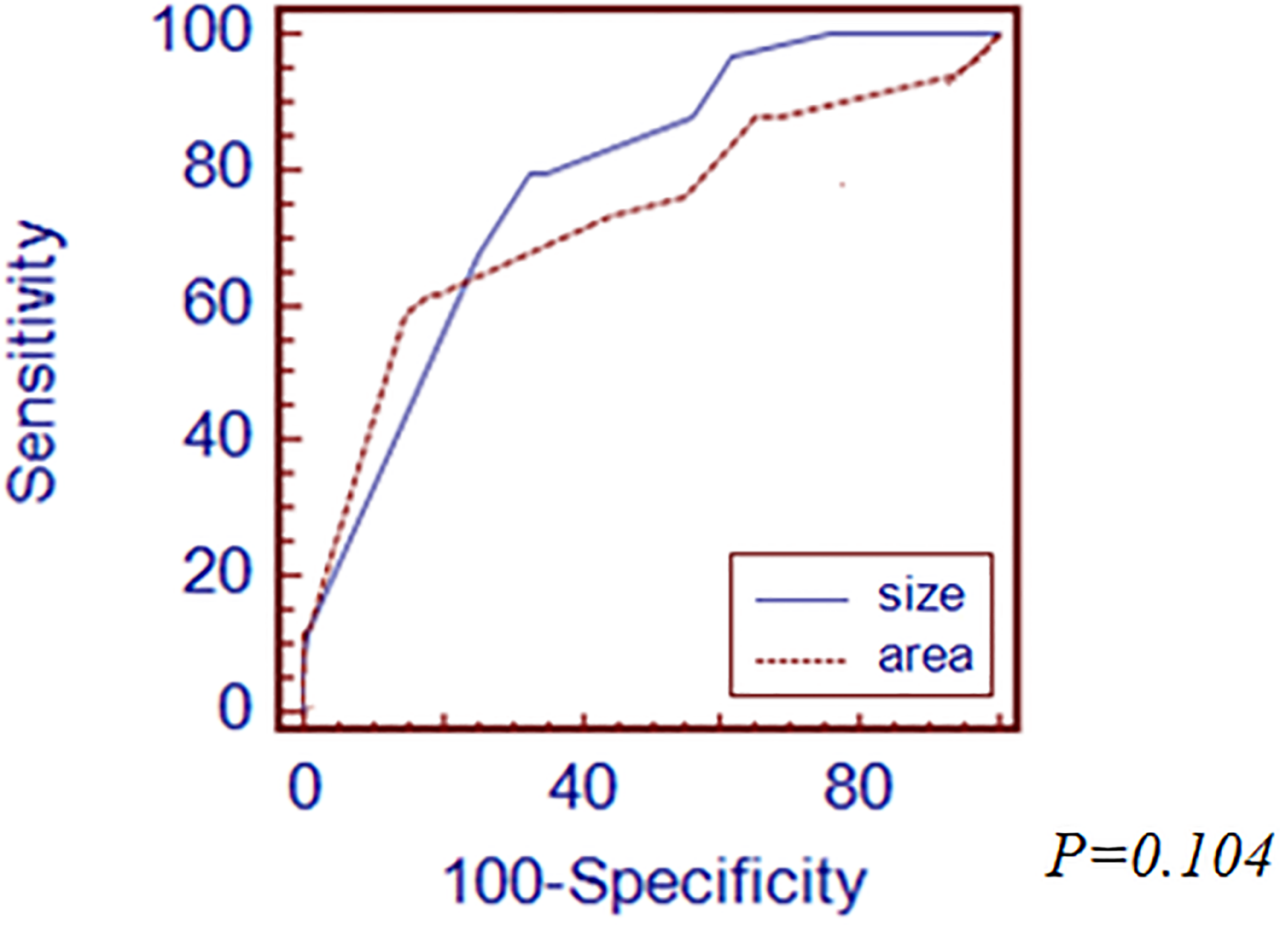
Comparison of ROC curves demonstrating the prediction power of LNM between the longest diameter and area of tumor in mucosal cancer.

**Fig 5:**
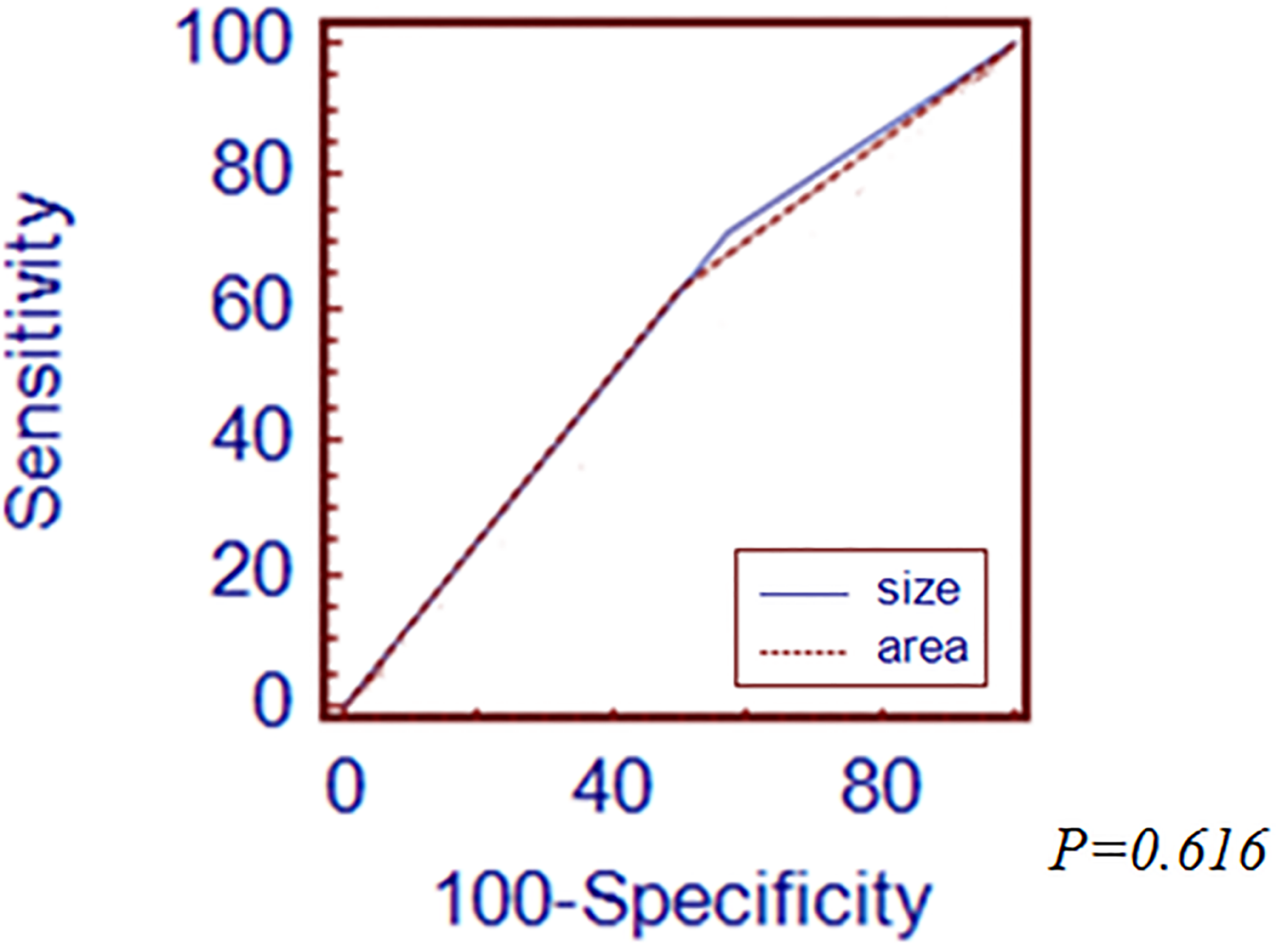
Comparing of ROC curves demonstrating the prediction power of LNM between tthe longest diameter and area of tumor in submucosal cancer.

First, we performed subgroup analysis according to histologic differentiation by Japanese classification. In differentiated type-EGC, the cutoff value was 16mm of the longest diameter and 216mm^2^ of tumor area. AUROC value of the longest diameter and tumor area was 0.863 and 0.866, respectively (Fig 2). There was no significant difference between the longest diameter and area of tumor for prediction of LNM. In undifferentiated type-EGC, the cutoff value of the longest diameter of tumor was 23mm and tumor area was 390mm^2^. AUROC value of the longest diameter of tumor was 0.841 and area was 0.836(Fig 3). There was no significant difference between the longest diameter and area of tumor. Secondly, we analyzed LNM prediction value between the longest diameter and area of tumor according to depth of invasion in EGC specimen. In mucosal cancer, the cutoff value was 15mm of the longest diameter of tumor and 495mm^2^ of tumor area. The AUROC value of the longest diameter of tumor was 0.780 and tumor area was 0.730 (Fig 4). There was no significant difference between the longest diameter and area of tumor for prediction of LNM. In submucosal cancer, the cutoff value was 20mm of the longest diameter of tumor and 400mm^2^ of tumor area. AUROC value of the longest diameter of tumor was 0.568 and tumor area was 0.564 (Fig 5). There was no significant difference between the longest diameter and area of tumor for prediction of LNM.

## Discussion

Even though 5-year survival rate of EGC exceeds 90%, LNM status is still the most important prognostic factor. Overall survival is worst in node positive compared to node negative EGC with 10 year survival rate of 72% and 92%, respectively [4]. ER is local treatment as standard curative treatment option for EGC. It is now widely performed in standard and expanded indications. So it is important the prediction of the clinical behavior of the tumor before performing ER for EGC. To predict clinical behaviors and prognosis of EGC, endoscopic gross appearance may be useful. Endoscopic gross appearance associated with histological differentiation and clinical behavior of EGC. LNM were significantly different according to the gross appearance of EGC[8].

Some previous studies reported that tumor size is known to be independent predictor of LNM[15, 16]. Katsube *et al*.[17] reported that in addition to submucosal invasion ≥0.5 mm, a diameter ≥ 30mm was a risk factor for LNM. Generally, maximal diameter of the tumor was used to evaluating the longest diameter of tumor. In the ER indication and other guidelines of EGC, one-dimensional diameter is commonly used[10]. However, the tumor shape of EGC is usually oval or round, some studies represented measuring tumor volume of EGC is associated with LNM [11, 18]. Jeon et al. [19] suggested calculating tumor area as 3.14 x 0.25 x maximal diameter x short diameter. In our study, we used multiplying the longest diameter and its perpendicular short diameter of the tumor in a surgical specimen to express the tumor area roughly. We added short diameter to conventional maximal diameter of tumor, represent tumor area as maximal diameter x short diameter.

When we analyzed of LNM prediction value between the longest diameter and area of tumor in total EGC, there is no significant difference between the longest diameter and area of tumor for prediction of LNM. We performed subgroup analysis in histologic type (Japanese classification), but there is no significant difference between the longest diameter and area of tumor for prediction of LNM. We also performed subgroup analysis according to the depth of tumor invasion, there is no significant difference between the longest diameter and area of tumor. Generally, the bidirectional growth of the tumors was more common tumor biologically, it could be related that the longest diameter of tumor, used in current ER indication, is sufficient to reflect tumor burden.

Recently, Kim et al. [20]suggested that tumor size using 2-dimensional method was significantly useful to predict for LNM in differentiated minute submucosal cancer. However, when we also analyze LNM prediction value between the longest diameter and area among differentiated minute submucosal cancers, there was no statistically significant difference Although our study concluded no statistical significance between the longest diameter and area of tumor, our study is the meaningful study to represent the tumor burden of EGC using tumor area calculated by multiplying the longest and short diameter through large numbers of the patients with EGC. Furthermore, our results suggested that the maximal diameter used in current ESD criteria of EGC could be a useful indicator of tumor burden to predict LNM. However, further prospective studies with a large sample size may be necessary to determine the role of tumor area for LNM prediction precisely.

In addition, our study has some limitations. First, there could be a selection bias because our study evaluated patients only underwent surgery and not included those underwent ESD. However, although ESD was performed based on endoscopically determined longest size of tumor, curative resection or non- curative resection was determined by pathological review after ESD. Therefore, to predict the possibility of LNM, we considered being sufficient using surgical specimen after gastrectomy for EGC. Second, by the limitation of retrospective analysis, a prospective analysis of undergoing ESD for EGC is necessary to confirm of the role of tumor area.

In conclusion, tumor area may not be more helpful to predict LNM than one-dimensional longest diameter of tumor in EGC. Therefore, maximal longitudinal tumor size may be sufficient as an indicator of tumor burden in EGC.

## Supporting information

S1 Fig Analysis of the correlation between the longest diameter and area of tumor. (TIF)

S1 File The data file of our manuscript. (SAV)
