# Supplementary figures and images for: The longest diameter of tumor as a parameter of endoscopic resection in early gastric cancer: In comparison with tumor area
Source: PLoS One. 2017 Dec 20;12(12):e0189649. doi: 10.1371/journal.pone.0189649 (PMC5738054; doi:10.1371/journal.pone.0189649)

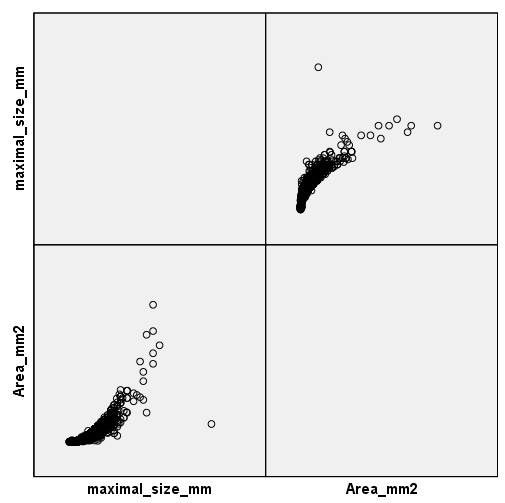

Supplement: S1 Fig — (TIF) [file pone.0189649.s001.tif]
